# MiR-539-3p impairs osteogenesis by suppressing Wnt interaction with LRP-6 co-receptor and subsequent inhibition of Akap-3 signaling pathway

**DOI:** 10.3389/fendo.2022.977347

**Published:** 2022-09-29

**Authors:** Alok Tripathi, Aijaz A. John, Deepak Kumar, Saurabh Kumar Kaushal, Devendra Pratap Singh, Nazim Husain, Jayanta Sarkar, Divya Singh

**Affiliations:** ^1^ Division of Endocrinology, Council of Scientific and Industrial Research-Central Drug Research Institute, Lucknow, India; ^2^ Academy of Scientific and Innovative Research (AcSIR) Ghaziabad, Uttar Pradesh, India; ^3^ Division of Cancer Biology, Council of Scientific and Industrial Research-Central Drug Research Institute, Lucknow, India

**Keywords:** microRNA, X-linked hypophosphatemia, PHEX, LRP-6, AKAP-3

## Abstract

X-linked hypophosphatemia (XLH), an inheritable form of rickets is caused due to mutation in Phex gene. Several factors are linked to the disease’s aetiology, including non-coding RNA molecules (miRNAs), which are key post-transcriptional regulators of gene expression and play a significant role in osteoblast functions. MicroRNAs sequence analysis showed differentially regulated miRNAs in phex silenced osteoblast cells. In this article, we report miR-539-3p, an unidentified novel miRNA, in the functional regulation of osteoblast. MiR-539-3p overexpression impaired osteoblast differentiation. Target prediction algorithm and experimental confirmation by luciferase 3’ UTR reporter assay identified LRP-6 as a direct target of miR-539-3p. Over expression of miR-539-3p in osteoblasts down regulated Wnt/beta catenin signaling components and deteriorated trabecular microarchitecture leading to decreased bone formation in ovariectomized (Ovx) mice. Additionally, biochemical bone resorption markers like CTx and Trap-5b were elevated in serum samples of mimic treated group, while, reverse effect was observed in anti-miR treated animals along with increased bone formation marker P1NP. Moreover, transcriptome analysis with miR-539-3p identified a novel uncharacterized Akap-3 gene in osteoblast cells, knock down of which resulted in downregulation of osteoblast differentiation markers at both transcriptional and translational level. Overall, our study for the first time reported the role of miR-539-3p in osteoblast functions and its downstream Akap-3 signalling in regulation of osteoblastogenesis.

## Graphical Abstract

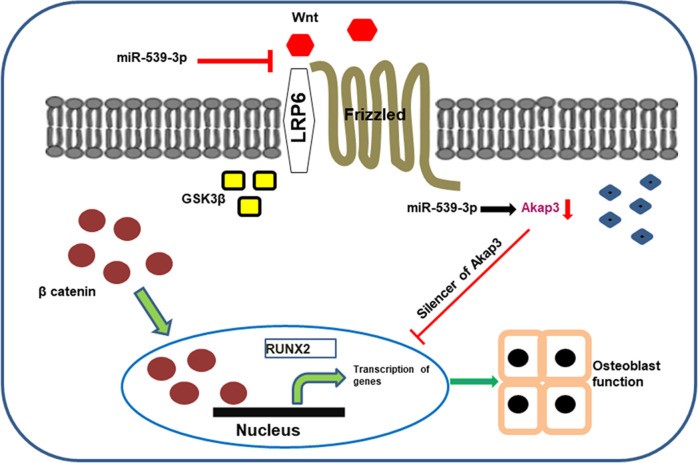

## Introduction

Rickets is a bone-growing disorder, characterized by abnormal mineralization of growth plates and bone tissue. Adults, when experience a similar condition, it is referred to as Osteomalacia. The most prevalent heritable form of rickets and Osteomalacia is X-linked hypophosphatemia (XLH). XLH, is a classical vitamin D–resistant disease in humans, characterized by renal phosphate (Pi) wasting with hypophosphatemia, defective bone and cartilage mineralization, aberrant vitamin D metabolism, dentine abnormalities, and stunted growth (1, 2). It has an incidence of approximately 1:20,000 live births. XLH due to a mutation in a phosphate-regulating gene with homologies to endopeptidases on the X chromosome (PHEX) is one of the most conventional genetic disorders of phosphate homeostasis (3). To date around 300 pathogenic Phex mutations have been reported (4), which have a dominant effect manifesting disease even in females. As the Phex gene is located on the X chromosome, there are 50% chance of an affected mother having children manifested with this condition. On the contrary, an affected father will pass the disease on to all of his daughters, but not to his sons.

Phex gene is often thought to be involved in the proteolytic degradation of extracellular matrix (ECM) proteins (5). In addition to this, it is capable of cleaving ASARM (acidic serine- and aspartate-rich MEPE-associated motif) peptide, a potent mineralization-inhibiting peptide from MEPE (Matrix extracellular phospho glycoprotein) and OPN (osteopontin), family of non-collagenous proteins and rescue mineralization inhibition assisted by phosphorylated ASARM as revealed by earlier reports have in *hyp* mice (6). Moreover, Phex is predominantly found to be expressed in bones and teeth as identified by studies of murine tissues and cell cultures, although *Phex* mRNA and/or protein have been found in the lung, brain, muscle, gonads, skin, and parathyroid glands, its expression in bone is restricted to osteoblast cells (7). Several studies have demonstrated that *Phex* mutation/deficiency results in FGF-23 overproduction, nevertheless the underlying mechanism responsible for overproduction of FGF-23 during *Phex* deficiency is yet unknown (8, 9).

In current years, a lot of focus is on aberrant microRNA (miRNA) expression in disease conditions like cancer and cardiovascular disorders. MiRNA is a tiny single-stranded non-coding RNA molecule found in plants, animals, and some viruses that regulates gene expression through RNA silencing and post-transcriptional modification. They are involved in a variety of biological and cellular processes, such as metabolism, differentiation, and apoptosis. Abnormal miRNA expression has been linked to the broad range of human illnesses and pathogenesis including cancer, diabetes, neurological disorders, pulmonary hypertension, heart failure, and autoimmune diseases due to dysfunction of their target genes (10). Several miRNAs have been discovered that either positively or negatively affect osteoblast development or bone formation by targeting critical osteogenic factors (11, 12). Translational studies suggest that miRNA signature may be useful in designating and forecasting the course of an increasing number of human pathologies.

Hence, in this study, we decided to investigate the role of signature microRNA in osteoblast cells transfected with Phex siRNA and their affect in the process of osteogenesis. The differential expression of numerous miRNA candidates was discovered during miRNA profiling of Phex-silenced osteoblast cells, the majority of which were up-regulated. More than eightfold upregulation in levels of miR-539-3p was found among the elevated miRNA candidates. Previous studies by different groups have shown the anti-tumor effect of miR-539-3p (13, 14). There is a report which also describes that miR-539-3p suppresses chondrogenic differentiation by targeting Sox-9 (15) but its function in osteoblasts remains uncharacterized. Thus, here we report the role of miR-539-3p in osteoblastogenesis and how it regulates bone formation. Further, we have elucidated the role of A-kinase anchor protein 3 (AKAP-3), a protein with uncharacterized role in bone, which was found be down regulated when a transcriptome analysis of osteoblasts overexpressing miR-539-3p was carried out.

## Material and methods

### Mouse calvarial osteoblast culture

Calvarial osteoblasts were harvested from cell culture obtained from 1-2 day old neonatal mice pups in accordance with standard protocols (16). Calvariae were surgically excised and then exposed to five successive digestions in 0.1% dispase (Sigma, St. Louis, MO, USA) and 0.1% collagenase P (Sigma, St. Louis, MO, USA) solution at 37°C. Cells released from the second to fifth digestions were collected, centrifuged, resuspended and plated in T-25 cm^2^ flasks in osteoblast growth medium containing α-MEM media (Sigma, St. Louis, MO, USA), 10% fetal bovine serum (FBS, Gibco, Life technologies, USA) and 1% penicillin/streptomycin (Sigma, St. Louis, MO, USA).

### Transfection assay

MCOs were seeded 1 day prior to the transfection in cell culture plates in osteoblast growth medium. Cells at 60-70% confluence were transfected with oligo miRNAs like miC (negative control), miR-539-3p (mimic) and anti-miR-539-3p (inhibitor) and siRNAs purchased from Thermo Fisher Scientific, USA by using lipofectamine 2000 (Thermo Fisher Scientific, USA) transfection reagent in Opti-MEM^®^I reduced serum (Gibco, Life technologies, USA) medium as per manufacturer’s protocol. Sequences are given in **Supplementary Table 1**. After 6 h, the medium was replaced with osteoblast differentiation medium. Further analysis was done by harvesting cells at different time intervals after transfection.

### Alkaline phosphatase assay

ALP activity was measured by trysinization of MCO at 70-80% confluence followed by seeding in 96 well (3000 cells/well) plates in osteoblast growth medium. Cells were then transfected with miR-539-3p, anti-miR-539-3p and negative control (miC) for 6h and changed with differentiation medium containing 10 mM beta-glycerophosphate (Sigma, St. Louis, MO, USA) and 50 μg/ml ascorbic acid (Sigma, St. Louis, MO, USA) for 48h (16, 17). Total ALP activity was evaluated by measuring absorbance at 405 nm using p-nitrophenylphosphate as a substrate (PNPP, Sigma, St. Louis, MO, USA).

### Mineralization assay

For mineralization studies, murine osteoblasts were cultured according to our previously published lab protocol (18, 19). Cells were seeded in α‐MEM medium, supplemented with 10% fetal bovine serum, 50 μg/ml ascorbic acid, and 10 mM β-glycerophosphate) and transfected with miC, miR-539-3p and anti-miR-539-3p. Cells were cultured for 14 days at 37°C in a humidified CO_2_ incubator and media was changed every 48 h. For *ex-vivo* after mineralization femoral bone marrows of autopsied Balb/c female mice of different groups were cultured in α‐MEM medium, supplemented with 10% fetal bovine serum, 10^-7^ M dexamethasone, 50 μg/ml ascorbic acid, and 10 mM b-glycerophosphate) and seeded in six‐well plate with a density 2 x 10^6^ cells/well and cultured for 21 days (20). After completion of time, the cells were fixed with 4% para formaldehyde (PFA) for 30 min at room temperature and stained with 40 mM alizarin red S, which stains nascent calcium and quantified with 10% Cetylpyridinium chloride (CPC) solution. The absorbance was taken at 595 nm on the next day (21).

### qRT-PCR analysis

Quantitative RT-PCR was employed to examine the mRNA expression levels. mirVana miRNA isolation kit was used for miR-539-3p quantification along with TaqMan miRNA reverse transcription kit (Thermo Fisher Scientific, USA) and a TaqMan miRNA assay kit (Thermo Fisher Scientific, USA). Total RNA was isolated from the cultured cells as per manufacturer’s instructions (mirVana miRNA isolation kit (Ambion, Carlsbad, CA, USA). TaqMan miRNA reverse transcription kit was used to create cDNA. 100 ng of total RNA was added to the reaction mixture, which included 10 RT buffer, Multi Scribe reverse transcriptase, 5 RT primers, 100 mM dNTPs, RNase inhibitor and nuclease-free water. Total RNA was isolated from the confluent cultured cells utilizing TRIzol (Life Technologies) for mRNA, while 2 μg of total RNA was used to synthesize cDNA using the High-Capacity cDNA Reverse Transcription Kit (Applied Biosystems™, Thermo Fisher Scientific, USA) in accordance with the manufacturer’s instructions. Expression analysis of several genes and miRNA by qRT PCR was carried out in accordance with previously reported protocol (22). **Supplementary Table 1** depicts the list of sequences of sense and antisense oligonucleotide primers. For triplicate reactions, the mean Ct (cycle threshold) results of each sample were calculated. The relative expression levels of miRNA and mRNAs were calculated using log2|2—DCt|, where DCt was measured by subtracting the Ct value of the target miRNA and mRNA from the Ct value of the internal control U6 (Cat no. 4427975) and GAPDH.

### miRNA target prediction and Luciferase reporter assay

The possible target genes for miR-539-3p were identified by employing two different target prediction tools, i.e., Target Scan and mirdb. 200 ng of empty pEZX-MT06 control clone (Genecopoeia Medical Center Dr. Suite 101 Rockville, MD 20850 USA) and 3′ UTR of LRP-6 clone were transfected for 6 h in Opti-MEM^®^I reduced serum medium with lipofectamine 2000 reagent. Cells were co-transfected with miR-539-3p or miC at concentrations of 50 nM. Renilla luciferase activity was used for normalisation and as an internal control for transfection efficiency. A dual-luciferase Reporter Assay System (Promega, Madison, WI 53711 USA) was used on a FLUOstar galaxy to quantify firefly and renilla luciferase in cell lysates. The activity of Renilla luciferase was utilised as a normalizer and an internal check for transfection efficiency.

### Transcriptome analysis

Confluent MCOs were transfected with or without miR-539-3p and total RNA was isolated. The integrity and quality of the isolated RNAs were tested by Agilent 2100 bio analyzer and the samples with RNA integrity score >7 were used for library preparation. NEBNext poly (A) mRNA magnetic isolation module (E7490S) was used for enrichment of mRNA using oligodT based beads. 100 ng of RNA was taken as input for the library preparation. RNA libraries were prepared as instructed in the manual of NEBNext Ultra II RNA library prep kit (E7775). Sequencing was performed on Illumina Hiseq 2500 next generation sequencing platform.

For data analysis, RNA-seq data were mapped to the mouse genome constructs using TopHat (v2.0.8b, http://tophat.cbcb.umd.edu/) (UCSC). The low-quality reads were eliminated from the raw sequencing data. Tophat (R software) was used to map the reads, and HTSeq (http://www-huber.embl.de/users/anders/HTSeq/doc/overview.html) was used to count the reads. The DESeq R software package was used to examine differentially expressed genes. The differentially expressed genes were discovered using Benjamini-Hochberg multiple testing adjustments.

### Lentiviral transduction

For transient shRNA transductions, 3T3 cells were seeded in a 6 well plate at a density of 0.2×10^6^ and grown overnight in a CO_2_ incubator. The cells were then transduced with concentrated lentiviral particles diluted in fresh Dulbecco’s Modified Eagle Medium-high glucose (DMEM-HG), using cationic polymer polybrene at a final concentration of 10μg/ml. At 72h post-transduction, cells were harvested and processed for immunoblotting to check the knockdown efficacy of the target gene.

For MCO study, cells treated with concentrated lentiviral particles with polybrene for 24h. After 24h, medium was changed with osteoblast differentiation medium. At 48h post-transduction, cells were harvested and processed for further analysis. Details of lentiviral vector and production of lentiviral particles has been given in supplementary section.

### Protein extraction and western blot analysis

Immunoblotting was done to check the expression levels of osteogenic proteins as well components that are involved in the Wnt/beta catenin pathway. For this purpose, cultured MCO cells were transfected with different oligo miRNAs and si-RNAs and incubated for 48h in the differentiation medium. Whole cell lysate was extracted with mammalian cell lysis buffer comprising protease inhibitor cocktail and phosphatase inhibitor (Sigma, St. Louis, MO, USA). Protein concentration was estimated by BCA assay, and then separated on different percentage of SDS-PAGE, which were then electroblotted onto PVDF membranes (Immobilon-P, Millipore, Billerica, MA, USA). The membrane was then probed with Runx-2, Type I collagen, Akap-3, Wnt-3a, LRP-6, β-Catenin, P-β-catenin, Lef-1 and β-actin antibodies as primary antibodies and incubated with secondary antibodies conjugated with Horseradish Peroxide (HRP) at 4°C overnight. Antibody details were given in **Supplementary Table 2**. On the Image Quant LAS 4000 (GEHealthcare) gel doc imaging system, Immunodetection was performed with an enhanced chemiluminescence kit (Immobilon-P, Millipore, Billerica, MA, USA). *ImageJ* software was used for the quantification of blots.

### *In vitro* osteoclastogenesis from mouse bone marrow cells

*In vitro* Osteoclastogenesis was performed as per standard protocol (19). In brief, bone marrow cells (BMCs) collected from the femurs and tibias of 8 to 9-week-old female BALB/c mice were allowed to proliferate by incubating in a T-25 cm^2^ flask using complete cell culture mix including α-MEM, 10% FCS, 1% penicillin/streptomycin, 10 ng/ml MCSF. Following overnight incubation, the non-adherent BMCs were plated at a density of 2 x 10^5^ cells/well in 48-well plates and then cultured in osteoclast medium containing 50ng/ml RANKL and 30ng/ml MCSF for 6 days by replacing the media after every 48h. The cells were then washed with PBS and fixed in 4% paraformaldehyde for 20 min. After that, tartrate-resistant acid phosphatase cells were stained according to the standard procedure (23). For transfection, at 60–70% confluence, mouse osteoclast cells were transfected with miC and miR-539-3p at a 50 nM concentration using RNAi Max (Invitrogen). After 48h of transfection, cells were isolated in order to measure the expressions of TRAP and RANK by employing real-time PCR and stained for TRAP positive cells (24).

### Ethics approval and animal studies

All animal care and experimental methodologies have been approved in advance by the Institutional Animal Ethical Committee (IAEC) at the CSIR-Central Drug Research Institute in Lucknow, India. Animal experiments were carried out in accordance with the guidelines provided by the Committee for the Control and Supervision of Experiments on Animals of the Indian Government (CPCSEA). This study’s animal research and laboratory techniques have been approved by the IAEC (approval no. CDRI/IAEC/2019/49/Dated-04/01/2019). In all the experiments, six-week-old female Balb/c mice were utilized. Animals were ovariectomized with general anesthesia employing dorsal approach and left for 1 month to develop osteopenic condition. Animals were randomly distributed in four groups, one with Sham+PBS (ovary intact), Ovx+miC, Ovx+miR-539-3p and Ovx+anti-miR-539-3p. The three ovariectomized groups were subcutaneously injected (one injection/week) for three weeks with miC, miR-539-3p and anti-miR-539-3p respectively. 100 μl of oligonucleotide mixture containing 5 μg of oligonucleotide with N/P ratio of 8, i.e., 0.16 μl of *in vivo*-jetPEI^®^ (Cat. No.201- 10G, polyplus-transfection, Illkirch, France) per ug of oligonucleotide was given to each mouse *via* subcutaneous injection behind the head region. On the fourth week, the mice were sacrificed and femur bones as well as serum samples were collected for analysis of trabecular microarchitecture using μCT and other biochemical parameters (16).

### Micro-CT analysis

The Sky Scan 1076 CT scanner (Aartselaar, Antwerp, Belgium) was used to perform micro-computed tomography of the femur bones as previously described (17). Briefly, the trabecular regions of bone samples were scanned with an X-ray source of 70 kV and 100 mA at a nominal resolution (pixels) of 18 mm. The image slices captured with 100 projections at 180° angular range were then reconstructed by utilizing the Sky Scan Nrecon software, employing a modified Feldkamp method that aids in the reconstruction of a distributed network. Several trabecular bone parameters were calculated.

### Expression of osteoblast markers in bone

Muscles were removed from collected bones from various groups, and bone marrow was flushed. Femur and tibia that had been cleaned were combined and pulverized in liquid N2. Total RNA was extracted using the frozen powder that had been placed into a tube containing Trizol followed by collected and qPCR analysis as described above. **Supplementary Table 1** represents the list of primer sequences.

### Measurement of bone-relevant serum parameters

In order to assess the biochemical parameters, blood serum was isolated from all the animal groups, and centrifuged at 4,000 rpm for 30 min. Serum levels of amino-terminal propeptide of type 1 procollagen (serum P1NP, Elabscience Biotechnology Inc., Donghu Hi-Tec Development area, Wuhan, Hubei,China), C-terminal telopeptide of type I collagen (serum CTx, Elabscience,Wuhan,China) and TRAP-5b (immunodiagnostic systems Holdings Ltd. UK) were determined by enzyme-linked immunosorbent assay (ELISA) kits following the manufacturer’s protocols.

### Bone histomorphometry

The forelimb was disarticulated through the knee joint and then decalcified using a decalcifying buffer (decalcifying solution lite, Sigma, USA) for 72 h. Thereafter, the femur’s epiphyseal region was treated with 70% isopropanol before being preserved in paraffin for sectioning. The representative pictures were taken after the staining of transverse sections (5 *μ*m) with hematoxylin and eosin (Sigma-Aldrich (St. Louis, MO) on EvosFL Auto (Life technologies) microscope. Mature osteoblasts were visible as a single layer of cuboidal or polygonal cells with eccentrically located nuclei on bone surface. The osteoblasts number per bone surface below the growth plate was calculated by tracing the section image onto a digitizing platform with the aid of a cameral lucida attachment and bioquant analysis software according to standard protocols (25).

### Statistics analysis

The data is presented as mean ± S.E.M. One-way ANOVA was used to analyze the data acquired from experiments with multiple treatments followed by multiple comparison Turkey test of significance using Graph Pad Prism version 7.04 software. In trials with only two treatment conditions, the two-tailed t-test was utilized to investigate statistical significance. All experiments were replicated thrice and representative experiments as mentioned.

## Results

### Silencing of PHEX gene inhibits osteoblast differentiation

**Figure 1:**
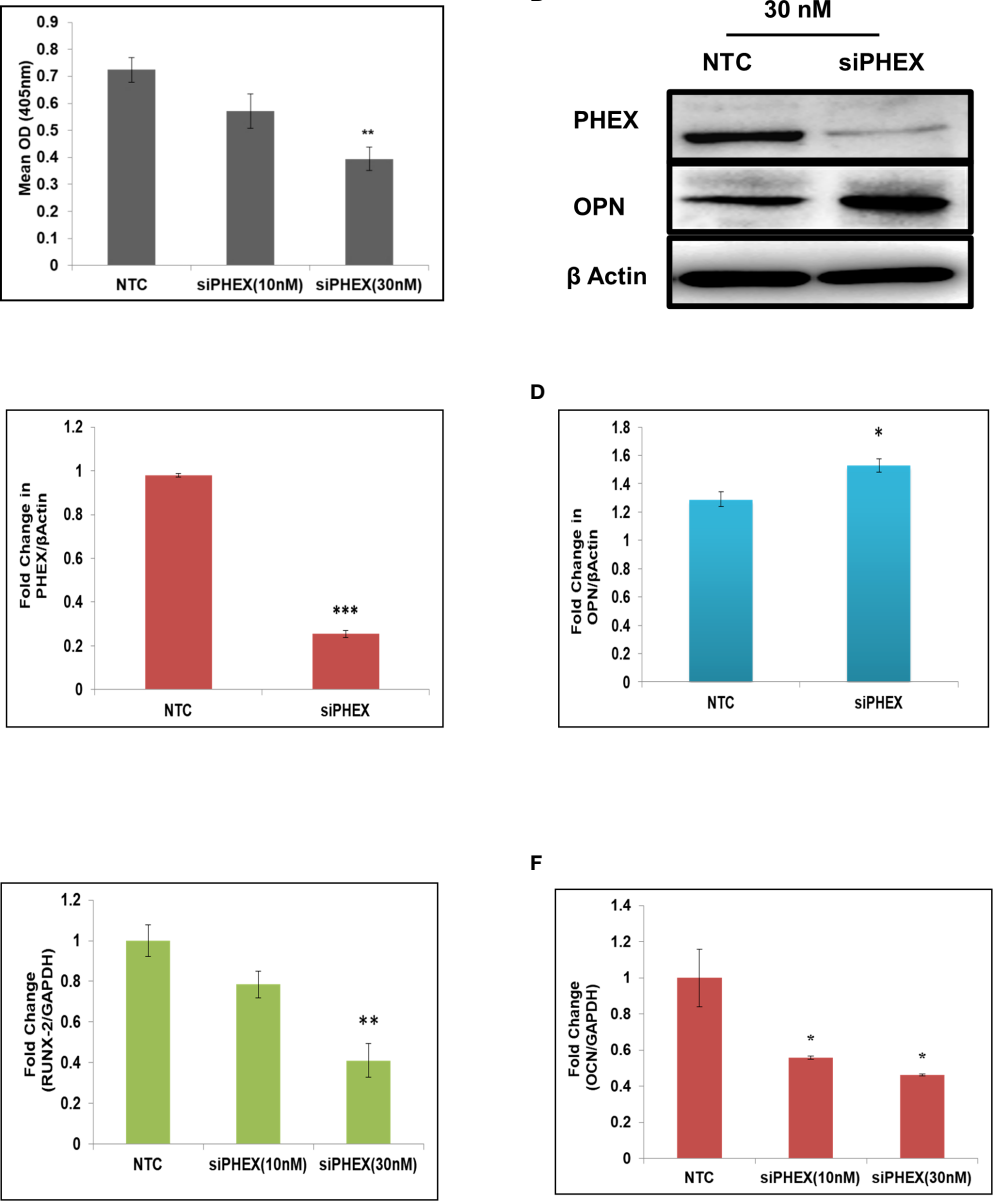
Silencing of *phex* inhibits osteoblast differentiation. **(A)** Murine osteoblasts transfected with different concentrations of silencer of *phex* and ALP activity measured at 48 h. **(B)** Western blot analysis of *phex* and OPN. **(C, D)** The relative expression of *phex* and OPN were quantified densitometrically by using *ImageJ* software. **(E, F)** qRT-PCR evaluation of osteogenic markers (RunX-2, and OCN) at 48 h. GAPDH was taken as internal control. The data is presented as mean ± SEM (n=6) *P < 0.05, **P < 0.01 and ***P < 0.001compared to NTC (scrambled siRNA).

Analysis of ALP activity, a marked osteoblast differentiation marker, after transfection of mouse calvarial osteoblasts with different concentrations of phex silencer was performed in order to examine the role of *phex* gene in osteoblast differentiation. The activity was found to be significantly inhibited at 30 nM concentration.

(**Figure 1A**) which implied that silencing of Phex gene resulted in suppression of osteoblast differentiation. This RNAi mediated silencing of phex as well as over-expression of osteopontin (OPN) in phex treated osteoblast cells were further confirmed by immunoblotting (**Figures 1B–D**). Moreover, RT-PCR data revealed a remarkably downregulated expression levels of osteogenic markers in *phex* silenced osteoblasts in comparison to non-target control (NTC, scrambled siRNA) treated cells (**Figures 1E, F**). This overall suggested that *phex* gene has an essential importance in contributing towards regulation of osteoblastogenesis.

### Identification of differentially regulated miRNA after *phex*-silenced osteoblast differentiation

**Figure 2:**
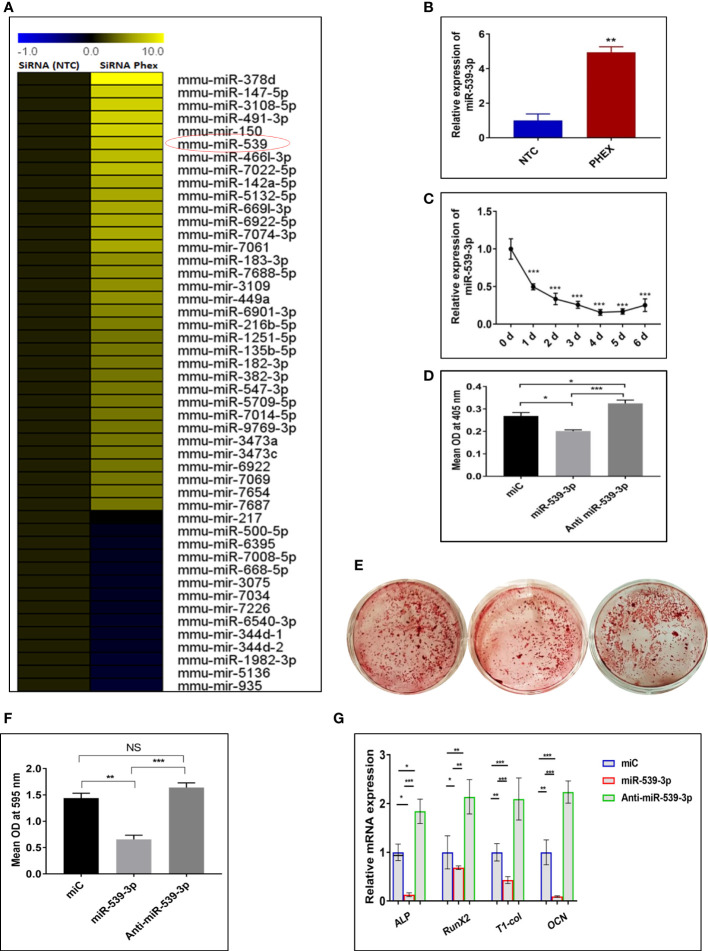
miRNAs expression profiles during *phex*-silenced osteoblast differentiation. **(A)** miRNAs sequencing profiling. Only representative miRNAs that were notably downregulated and upregulated are given, with yellow denoting high expression and blue indicating low expression respect to the median (n=2). **(B)** miR-539-3p expression in siPHEX osteoblast cells after 48 h of transfection. Data are expressed as mean ± SEM (n=6), **P < 0.01 compared NTC group. **(C)** Changes in miR-539-3p expression during osteoblast differentiation with the time. **(D)** Mouse calvarial osteoblasts (MCOs) were treated with negative control (miC), miR-539-3p and anti-miR-539-3p in osteoblast differentiation medium and ALP activity was measured after 48 h. **(E)** Murine osteoblasts were seeded in 6-well plates and cultured for 14 days followed by alizarin red S staining. Representative photomicrograph show mineral nodule formation in different groups. **(F)** Quantification of alizarin red S staining at 595 nm. **(G)** qRT-PCR evaluation osteogenic marker genes (ALP, RUNX-2, Type-1-collagen, OCN normalized to GAPDH) at 48 h. Data are expressed as mean ± SEM (n=6) *P < 0.05, **P < 0.01, ***P < 0.001 and NS, non-significant compared in between groups.

As silencing of Phex resulted in suppressed osteoblast differentiation, calvaria-derived mouse osteoblast cells were subjected to next generation microRNA analysis post transfection with or without siRNA of *phex* gene. MicroRNA analysis revealed that *phex* silencing resulted in up regulation of several miRNAs, while; only a few were found to be downregulated. Among them, miR-539-3p, was shown to exhibit 8.5-fold increase in expression during phex-silenced osteoblast development (**Figure 2A**). Though, miR-539-3p is often recognized as a tumor suppressor, it has never been investigated for its functional role in osteoblast differentiation. Quantitative real-time PCR (qRT-PCR) in murine osteoblast cells was used to further confirm the miRNA data analysis which showed ~5-fold upregulated expression of miR-539-3p in siPhex treated osteoblast cells (**Figure 2B**), whose expression was found to be decreased during the course of osteoblast differentiation (**Figure 2C**).

### miR-539-3p regulates osteoblast differentiation

The effect of miR-539-3p on osteoblast differentiation was evaluated, by transfecting osteoblast cells, with 30 nM of miR-C, 50 nM of mimic miR-539-3p and 30 nM of inhibitor anti-miR-539-3p, in growth medium with 10 mM b-glycerophosphate and 50μg/ml ascorbic acid followed by measurement of ALP activity after 48 h. miR-539-3p-transfected cells demonstrated significantly diminished ALP activity when compared to miR-C transfected cells, while, in anti-miR-539-3p-transfected cells, this effect was found to be reduced (**Figure 2D**). Following this, the effect of miR-539-3p was seen on mineral nodule formation. As shown in **Figures 2E, F** transfection of miR-539-3p decreased mineralized nodule formation in 14-day culture. However, anti-miR-539-3p treatment significantly increased the mineral nodule formation. In addition to this, inhibition in expression of certain osteogenic gene markers like ALP, RUNX-2, Type I col, and OCN was found to be mediated by mimic miR-539-3p (**Figure 2G**). Contrary to this, increased expressions of these markers were found in anti-miR-539-3p-transfected cells. These results thus suggested that osteoblast differentiation is negatively regulated by miR-539-3p.

### miR-539-3p directly targets LRP-6

**Figure 3:**
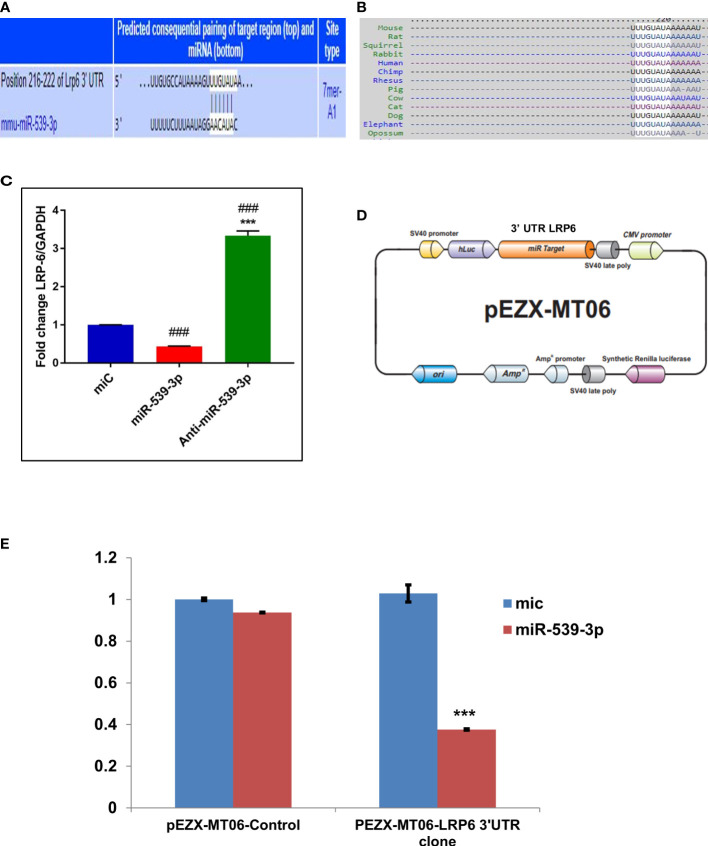
miR-539-3p target genes identification and its validation. **(A)** The complementarity sequences of miR-539-3p to the 3’ UTR of LRP-6 were evaluated using computational analysis. **(B)** The sequence is consistent across species. **(C)** The miR-539-3p expression plasmid or a control was transfected into osteoblasts. LRP-6 expression was determined using RT-PCR. GAPDH was employed as an internal control. All data represent mean ± S.E. (n = 6). ***P < 0.001 compared with the control, ^###^P< 0.001 compared with miR-539-3p. **(D)** Schematic presentation of the reporter plasmid used to demonstrate the impact of LRP-6 3’ UTR on luciferase activity. **(E)** miR-539-3p over-expression’s effect on a dual luciferase reporter plasmid carrying the LRP-6 3’ UTR was analyzed. Cells were co-transfected with miR-539-3p or miC and either the pEZXMT06 control or LRP-6 3’UTR clones. In cell lysate, firefly and renilla luciferases were quantified.

Further investigation to understand the possible mechanism by which miR‐539‐3p affect osteoblast differentiation was done by employing target prediction programs such as Targetscan (http://www.targetscan.org/cgi‐bin/search.cgii) and mirdb (http://www.mirdb.org/cgi‐bin/search.cgi). This revealed several target genes. Amongst these, LRP‐6 was chosen due to its important role in Wnt signaling. Besides, it possessed two sequence similarity sites, having 7‐mer A1 and 8-mer associations with miR‐539‐3p in the seed sequence and conserved sequence in different species (**Figures 3A, B**). In order to confirm that LRP-6 is the specific target of miR-539-3p, mRNA levels of LRP-6 was assessed in murine osteoblast cells transfected with miC or mimic or inhibitor. LRP-6 mRNA expression was shown to be dramatically reduced in cells transfected with miR-539-3p whereas, the same was found to be reversed in anti-miR-539-3p-transfected osteoblasts (**Figure 3C**). Further, to confirm the specificity of target, two luciferase reporter constructs were prepared, one without 3′‐UTR (control/empty clone) and other including 3′‐UTR LRP‐6. In osteoblast cells, these clones were co-transfected with miR-539-3p or miC, and luciferase activity was monitored. Co-transfection of miR‐539‐3p with 3′‐UTR of LRP-6 suppressed luciferase activity of reporter gene, However, the activity was found to be unaffected upon co-transfection of miR‐539-3p with empty vector/control clone (lacking 3′‐UTR), thereby confirming the target specificity (**Figures 3D, E**).

### miR-539-3p regulates Wnt/Beta catenin pathway thereby inhibiting osteoblast differentiation

**Figure 4:**
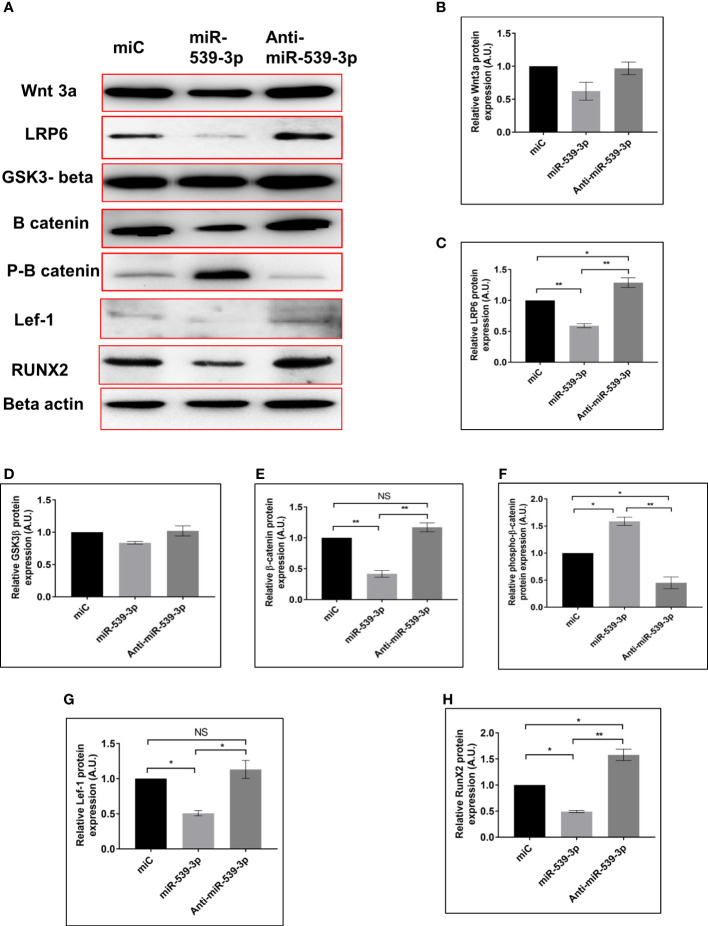
miR-539-3p inhibits osteoblast differentiation by regulating the LRP-6 wnt/beta catenin signalling pathway. **(A)** Cell lysate was obtained 48 h after transfection with miC, miR-539-3p, and anti-miR-539-3p, and immunoblotting of Wnt3a, LRP-6, GSk3beta, catenin, P-catenin, Lef-1and RunX-2 was done. As an internal control, beta actin was employed. **(B–H)** The relative expression of proteins was quantified densitometrically by using *ImageJ* software. All data represent mean ± SE (n = 3). *p < 0.05, **p < 0.01 and NS, non-significant compared between miC. miR-539-3p and anti-miR-539-3p conditions.

To investigate the impact of miR539-3p on Wnt signalling elements, miR‐539-3p was over-expressed in osteoblast cells. In order to examine its effect on Wnt signaling components as it directly targets LRP-6 (a known activator of Wnt-β catenin pathway). Cells were also transfected with miC (negative control) and inhibitor (anti‐miR‐539‐3p). Antibodies specific for the Wnt signalling pathway were used to probe the protein lysate obtained from transfected cells. Protein expression of Wnt3a, LRP-6, beta catenin, Lef-1 and RunX-2 were downregulated, as revealed by western blot analysis in miR-539-3p treated cells as compared to miC. Contrary to this, opposite result was obtained in cells transfected with anti-miR-539-3p (**Figures 4A–C, E, G, H**). Lesser effect on the expression of GSK3β was found (**Figure 4D**). Importantly, Phospho-beta catenin level was also found to be upregulated in miR-539-3p treated cells which was decreased in cells transfected with the inhibitor (**Figure 4F**). These results clearly showed that miR‐539‐3p inhibits osteoblast differentiation by suppressing the Wnt/beta catenin signaling.

### miR-539-3p promotes differentiation of osteoclast from bone marrow cells

**Figure 5:**
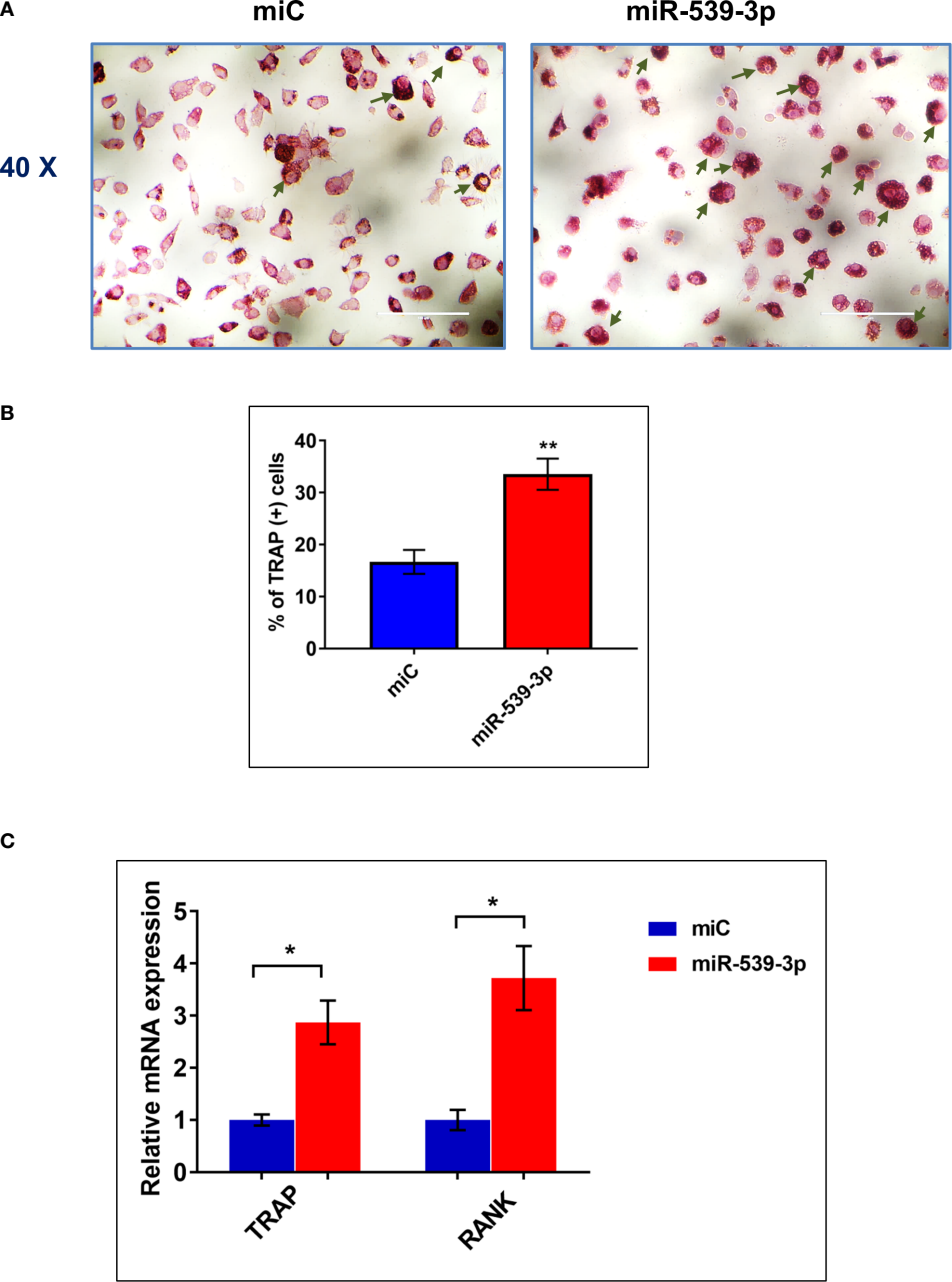
miR-539-3p induced osteoclasts differentiation **(A)** Representative photomicrograph (40x magnification) shows induction of osteoclastogenesis by miR-539-3p from BMCs in presence of M-CSF (30 ng/ml) and RANKL (50 ng/ml) in six days culture. **(B)** Quantitative analysis of TRAP^+^ cells from ten different areas at various treatment conditions. **(C)** Quantification of mRNA levels of TRAP and RANK gene, by qPCR analysis from the total RNA isolated from cultured cells. Data represents three independent experiments and expressed as mean ± SEM followed by the unpaired t-test of significance *p < 0.05, **p < 0.01 using Prism version 7.04 software.

After confirming the negative regulation of osteoblastogenesis by miR-539-3p, we have determined its direct role in osteoclastogenesis. **Figure 5A** depicts the representative image of TRAP positive cells in BMC cultures treated with or without miR-539-3p. The quantitative analysis indicated that ~34% TRAP positive cells were present in miR-539-3p treated cells in comparison to miC negative control, which was ~16% (**Figure 5B**). Moreover, miR-539-3p elevated the mRNA expression levels of RANK (P<0.05) and TRAP (P<0.05) as compared to miC negative control (**Figure 5C**). These data clearly indicated that miR-539-3p directly affects osteoclastogenesis by stimulating it.

### miR-539-3p directs bone formation *in vivo*

**Figure 6:**
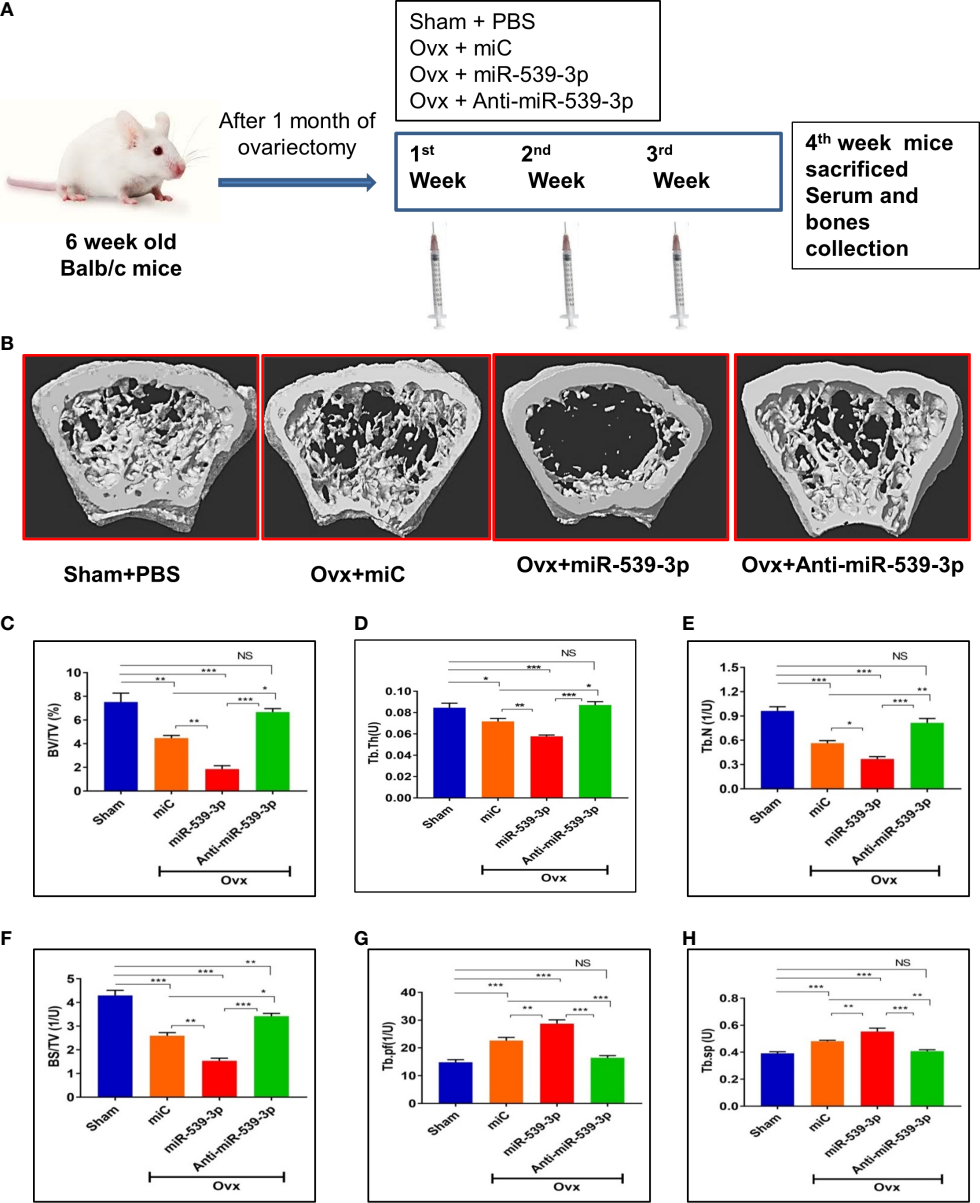
miR-539-3p suppresses trabecular bone formation. **(A)** Work plan of *in vivo* study. **(B)** Representative 3d microCT images of femur bone. **(C–H)** Quantification of microCT data revealed that anti-miR-539-3p treated mice showed notably enhanced bone parameters, including BV/TV, Tb. N, Tb.Th and BS/TV with a concurrent decrease in Tb.pf and Tb.Sp in femora, while in comparison to the miC group, these variables were reversed in miR-539-3p treated mice. All data represent mean ± SE (n = 6). *p < 0.05, **p < 0.01, ***p < 0.001 and NS, non-significant compared in between the groups.

To further investigate the functional role of miR-539-3p, *in vivo* studies were conducted which involved the use of chemically modified sense and antisense oligonucleotides specific to miR-539-3p. For this, animals were ovariectomized to instigate osteopenic condition. **Figure 6A** depicts the overall study plan. Subcutaneous administration of mice with readymade miRNA of miC, miR-539-3p and anti-miR-539-3p at a dose of 5μg/animal followed by treatment with oligonucleotides for three weeks was done, wherein; Sham+PBS (0.1 ml) served as controls. Micro-CT analysis of euthanized animals on the fourth week was performed to quantify the BV/TV (bone volume/tissue volume ratio), (Tb.N) trabecular number, (Tb.Sp) trabecular separation, (Tb.Th) trabecular thickness, (Tb.Pf) trabecular bone pattern factor and BS/TV (Bone surface/tissue volume). The representative micro-CT images are shown in (**Figure 6B**). Quantification of micro-CT data revealed that miR-539-3p inhibitor-treated Ovx mice showed a significant increase in bone parameters, including BV/TV, BS/TV, Tb.N and Tb.Th (**Figures 6C–E**), with a concomitant decrease in Tb.Pf and Tb.Sp in femora bones. However, reverse effects were observed in miR-539-3p treated Ovx mice (**Figures 6F–H**).

**Figure 7:**
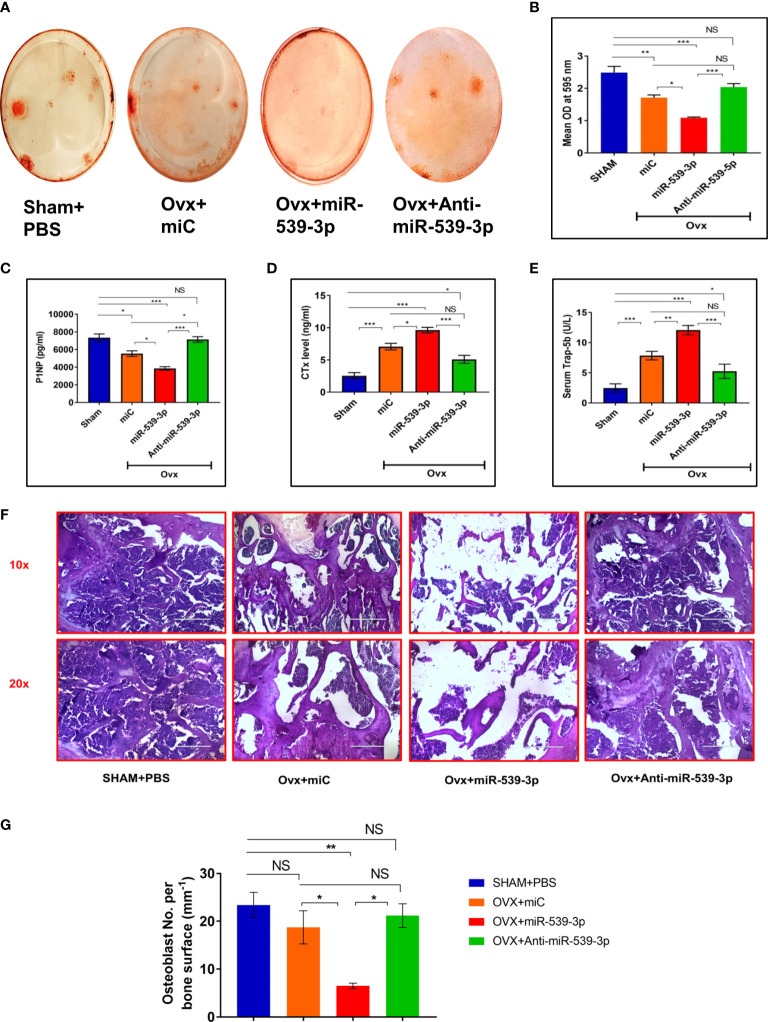
**(A, B)** *Ex vivo* mineralization representative images of femur bone and quantification. **(C)** Serum P1NP **(D)** Serum CTX-1. **(E)** Serum Trap-5b. **(F)** Representative HE images of different groups. **(G)** Quantification of osteoblast number per bone surface (n=3). All values represent means ± S.E. (n = 6). *P < 0.05, **P < 0.01, ***P < 0.001 and NS, non-significant compared in between the groups.

We also did *ex-vivo* mineral nodule formation efficacy in various groups. Less mineral nodule formation was seen in Ovx+miC (p < 0.01) group as compare to Sham+PBS. However, miR-539-3p treatment further decreased nodule formation (p < 0.001) in comparison to Sham+PBS. Opposite results were observed in anti-miR-539-3p treated group (p < 0.001) (**Figures 7A, B**). Further, osteogenic markers expressions were also assessed in bone samples of different groups. Transcript levels of T-1-col, RunX-2 and OCN were significantly decreased in miR-539-3p group. Opposite effect was found in anti-miR-539-3p group (**Supplementary Figure 2**).

Serum P1NP and CTx are considered as crucial bone formation as well as bone resorption markers, respectively. Hence, the ability of miR-539-3p to enhance bone resorption and suppress bone formation was analyzed. Consequently, a remarkable dip in P1NP serum levels was noticed in mice treated with miR-539-3p. This effect was totally reversed in mice treated with anti-miR-539-3p (**Figure 7C**). On the other hand, CTx levels were much higher in mice treated with miR-539-3p which returned back to the sham levels in anti-miR-539-3p treated animals (**Figure 7D**). Further, effect of miR-539-3p on osteoclastogenesis was also checked by evaluating serum levels of Trap-5b. Trap-5b is a well-known osteoclast marker and was found to be increased in miR-539-3p treated group. Its levels were decreased in anti-miR-539-3p treated animals (**Figure 7E**).

Furthermore, histological analysis performed by H&E staining technique exhibited scanty and thinning trabeculae, deprived of connectivity in miR-539-3p treated femur bones, whereas treatment with anti-miR-539-3p considerably guarded the trabecular bone and manifested eminent connectivity (**Figure 7F**). Moreover, the osteoblasts number per trabecular bone surface were found to be less in miR-539-3p treated ovx mice while in anti-miR-539-3p administered group osteoblasts number per bone surface were comparable to sham (**Figure 7G**).

Taken together, miR‐539‐3p regulates bone metabolism and its inhibitor furnished defense against bone loss in ovariectomized mice that are estrogen depleted and susceptible to bone loss.

### Differential expression analysis and validation of targets

**Figure 8:**
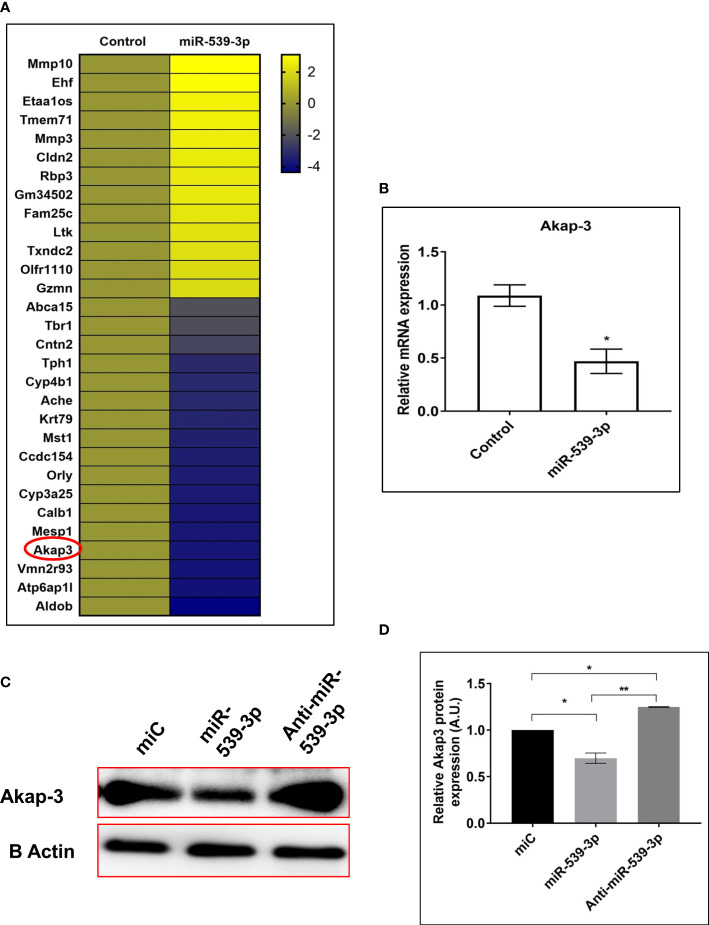
**(A)** Heat map of differentially regulated genes in control and miR-539-3p treated conditions (n=2). **(B)** RT-PCR validation of Akap-3 gene in control vs miR-539-3p transfected osteoblast cells. Data are expressed in means ± S.E. (n = 6). *P < 0.05 compared with control. **(C)** Western blot analysis of Akap-3 in miC, miR-539-3p and anti-miR-539-3p transfected cells after 48 h of transfection. **(D)** Densitometry of blots (n=3). Data are expressed as mean ± SEM (n=6) *P < 0.05 and **P < 0.01 compared in between groups.

Once, the role of miR-539-3p was ascertained in osteoblasts, we went on to study a transcriptome signature of MCO transfected with or without miR-539-3p. Based on the fold change (log2fold), depicting differences between miR-539-3p and control samples, a heat map of most downregulated as well upregulated genes in miR-539-3p conditions was generated. Genes with the lowest log2fold was denoted by blue color and largest log2fold with yellow color, respectively, with significant P value (**Figure 8A**).

Based on the analysis, 5 most upregulated and 10 downregulated targets were selected and their mRNA expressions were checked by qPCR for validation of the transcriptome profiling data (data not shown). Among the downregulated targets, Akap-3 expression was decreased in miR-539-3p treated cells as compared to control (**Figure 8B**). Further, protein expression of Akap-3 as revealed by western blot analysis was downregulated in mimic condition and restored in inhibitor treated cells (**Figures 8C, D**). Thus, downregulation of Akap-3 in miR-539-3p treated osteoblast cells might be indicative of its potential role in osteoblast functions.

### Loss of function of novel identified Akap-3 gene impairs osteoblast differentiation

**Figure 9:**
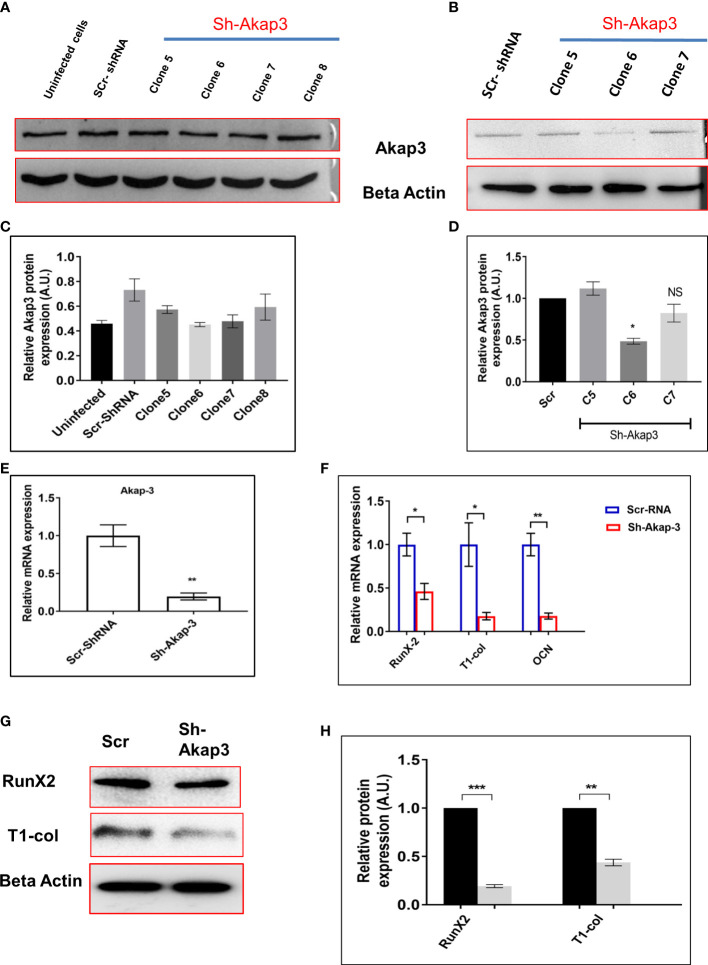
Effect of Akap-3 knock down on osteoblast proliferation. **(A)** Akap-3 protein level in 3T3 cells transduced with control shRNA or Akap-3 shRNA as determined by western blot using whole cell lysates. Data are expressed in mean ± SEM (n=3) *P < 0.05 compared with control scr shRNA **(B)** Western blot analysis of Akap-3 protein level in MCO cells shAkap-3 clones transduced with control shRNA. Values are expressed in mean ± SEM (n=3) *P < 0.05 compared with control scr shRNA. **(C, D)** quantification of blots by using *ImageJ* software. **(E)** m-RNA expression of Akap-3 gene in control vs shAkap-3 treated MCO cells. Values (mean ± SEM; n=4) **P < 0.01 compared with control scr-shRNA. **(F)** The expression of osteogenic marker genes (RunX-2, T-1-col and OCN) was evaluated in control and Akap-3 shRNA MCO treated cells using qRT-PCR. Data normalized with expression of internal control GAPDH. **(G)** The protein expression of osteogenic markers (RunX-2, T-1-col and OCN) was evaluated in control and Akap-3 shRNA MCO treated cells using western blots. **(H)** Densitometry analysis of blots. All values (mean ± SEM; n=3) *P < 0.05, **P < 0.01, ***P < 0.001, and NS, non-significant compared with control scr-shRNA.

Akap-3 gene was knocked down using lentivirus shRNA, in 3T3 cells to investigate its role in osteoblast differentiation. Different shRNA clones of Akap-3 in 3T3 cells were confirmed, out of which Clone 6 and clone 7 were found to be downregulated as compared to scrambled-shRNA control (**Figures 9A, C**). These clones were then transfected in calvarial derived osteoblast cells. Clone 6 showed significant down regulated expression confirming the gene knock down as compared to scr-shRNA control (**Figures 9B, D**). Furthermore, Akap-3 expression was significantly decreased at both the transcriptional as well as translational level, in cells expressing Akap-3 shRNA as compared to control shRNA (**Figure 9E**).

To further characterize the effect of Akap-3 on osteoblast differentiation, mRNA and protein levels of osteogenic markers like RunX-2, Type-1-col and OCN were assessed which demonstrated a downregulated expression in sh-Akap-3 cells as compared to scr-shRNA control (**Figures 9F–H**). This again implies that loss of Akap-3 gene function inhibits osteoblast differentiation. The same observations were obtained when siAkap-3 was transfected in osteoblast cells (**Supplement Figure 1**)

## Discussion

XLH is the most common form of heritable rickets resulting from Phex gene mutation and causes abnormalities of osteocyte and osteoblast function. Though Phex mutation results in XLH, but the underlying mechanism is not well known. As miRNAs have emerged as a major regulator of gene functions, and their expression is altered in diseased conditions, we decided to study the miRNA signature in osteoblast cells where Phex gene was silenced followed by miRNA profiling studies.

In the current study, we discovered a previously unknown function of miR-539-3p, which inhibits bone formation by suppressing LRP-6. We also discovered a novel function of miR-539-3p regulated A-kinase anchor protein 3 (Akap-3) in osteoblastogenesis which we report for the first time. Previous studies with miR-539-3p have reported of its tumor suppressor properties. Zhou et al. have reported that it inhibits proliferation and invasion of gastric cancer cells by targeting CTBP1 (14). Also Wang et al. have reported the anti-tumor effect of miR-539-3p on colon cancer (13). Though there is also a study where it is shown that by targeting Sox9, miR-539-3p inhibits chondrogenic development in human adipose stem cells (15), but there are no reports of its role in osteoblast differentiation and functions. Here, we show that miR-539-3p negatively regulates osteoblast functions by targeting LRP-6. We also identified a novel function for miR-539-3p regulated Akap-3 gene in osteoblastogenesis.

We started our study by confirming the observation that Phex silencing suppresses the osteoblast differentiation. Subsequently, osteoblast cells where Phex gene was silenced were subjected to miRNA profiling. A number of miRNAs were found to be differentially expressed. Owing to significant up-regulation of miR-539-3p, its tumor suppressive property and uncharacterized function in osteoblastogenesis, we decided to focus on this miR candidate. We thus sought to determine whether miR-539-3p directly regulates osteoblast differentiation. In cells overexpressing the miR-539-3p, there was a decrease in ALP activity and the expression of osteogenic markers (ALP, Type-1-col, RunX-2 and OCN).

Potential target genes were sought using target prediction tools like Target Scan and mirdb to study the molecular mechanism by which miR-539-3p influences osteoblastogenesis. The miR-539-3p gene was discovered to target a number of genes; however, one of the more intriguing targets for us was LRP-6, which is a crucial component of the wnt signalling pathway for osteoblast formation (26, 27). The observation in target prediction tools was corroborated by severe reduction in LRP-6 transcript levels in miR-539-3p transfected cells while more than 3-fold enhancement in expression of LRP-6 was obtained in anti-miR-transfected calvarial osteoblasts. Further, the luciferase reporter assay supported this result. We discovered that over-expression of mimic inhibited the reporter construct’s luciferase activity using a luciferase 3′ UTR-LRP-6 reporter gene. These observations were corroborated by protein expression studies where wnt components like Wnt 3a and LRP-6 were depleted in mimic transfected cells while anti-miR transfection significantly increased their protein levels. These results suggest that one of the mechanism by which miR-539-3p inhibits osteogenesis is by restraining the stimulation of Wnt signaling. Besides, miR-539-3p transfection in osteoclast precursor cells, led to elevated osteoclastogenesis. Thus, not only it suppressed osteoblastogenesis but also enhanced the process of osteoclastogenesis.

As all these observations were carried out in *in vitro*, we decided to study the impact of silencing miR-539-3p in an animal model. For the *in vivo* study, ovariectomized BALB/c mice were subcutaneously injected with a miR-539-3p mimic and its inhibitor. The Ovx mice model was chosen because it accurately mimics the critical clinical aspect of an estrogen-depleted human skeleton and therapeutic drug response. Our results showed that anti-miR-539-3p treatment resulted in considerable augmentation in trabecular microarchitecture in Ovx animals. In fact, anti-miR-539-3p supplementation induced significant enhancement of trabecular architecture in Ovx mice which was equal to sham group. Contrasting results were seen in mimic-treated groups. These data were strongly supported by increased trabecular connectivity, a reduction in serum CTx and Trap-5b levels in anti-miR treated groups. Importantly, serum P1NP, an established bone formation marker was significantly elevated in anti-miR group. Overall, data clearly demonstrates that miR-539-3p negatively regulates bone formation.

Though above results aptly depict the negative regulation of osteogenesis by miR-539-3p, we deemed it necessary to carry out a transcriptome profiling of miR-539-3p overexpressing osteoblast cells to further find out its detailed mechanism of action. Among the various targets, AKAP-3 was of particular importance. AKAPs are a group of structurally diverse proteins which bind to the regulatory subunit of protein kinase A (PKA) and confine the holoenzyme to discrete locations within the cell. A report by Koide et al. has shown that mice deficient in AKAP-13 are osteoporotic and have impaired osteogenesis (28). However, role of AKAP-3 is not reported in osteogenesis yet. A study by Dema et al. has ascribed a function to a cytosolic AKAP-PKA interaction as a regulatory factor in the control of canonical Wnt signaling (29). Also, Weivoda et al. have shown that wnt interactions with Fzd and Lrp5/6 co-receptors lead to activation of both canonical and cAMP/PKA signaling pathways (30). The cAMP/PKA subsequently activates AKAPs which then suppress NFATc1 thus blocking osteoclast differentiation. As miR-539-3p was also targeting wnt signaling pathway, we decided to study the role of miR-539-3p regulated AKAP-3 in osteogenesis. Akap-3 gene was knocked down using lentivirus shRNA and the clone was transfected in calvarial osteoblast cells. Cells transfected with shAKAP-3 exhibited significant reduction in ALP activity and osteogenic markers like RunX-2 and Type-1 collagen. In conclusion, we propose that miR-539-3p blocks stimulation of LRP-6 that may prevent the activation of cAMP/PKA/AKAP-3 pathway thus resulting in increased NFATc1 expression, enhanced osteoclastogenesis and reduced bone formation. Our results provide new biomarkers and therapeutic targets; miR-539-3p and AKAP-3 in bone disorders like XLH, Osteomalacia and Osteoporosis. However, additional studies are required to corroborate these findings due to certain limitations to our study. There are a number of predicted targets for miR-539-3p in Target Scan and miRdB algorithms. It’s unclear whether these unidentified target genes are involved in miR-539-3p-mediated osteoblast differentiation. Furthermore, because a single miRNA affects a large number of genes, the potential off target effects of miRNA therapeutics is a major area of concern. Stability of miRNA is another important factor. Thus, correct delivery systems will be required to specifically aim the candidate microRNA at specific target site which in this case is bone.

## Author’s note

The CDRI communication number for this article is 10456.

## Data availability statement

The original contributions presented in the study are included in the article/**Supplementary Material**. Further inquiries can be directed to the corresponding author.

## Ethics statement

The animal study was reviewed and approved by Institutional Animal Ethics Committee (IAEC), National Laboratory Animal Centre, CSIR-CDRI (Registration no.:CDRI/IAEC/2019/49/Dated-04/01/2019).

## Author contributions

DS and AT conceptualize, design the experiments and wrote the manuscript. AT performed most of the experiments and analyzed the data. AJ contributed in **Figure 1** experiment. DK and JS provided the knockdown clones and performed the experiments. SKK, DPS and NH helped in animal related experiments. All authors reviewed the manuscript carefully and agreed to the submission.

## Funding

This study was funded by the Department of Science and Technology under the project GAP303, Council of Scientific and Industrial Research under the projects MLP-2028 and MLP-2035, New Delhi. Authors are thankful to fellowship grants from Indian Council of Medical Research (AT, SKK and NH), University Grants Commission (DK), CSIR Council of Scientific and Industrial Research (DPS), New Delhi.

## Acknowledgments

Authors are thankful to Mr. G.K. Nagar for his technical assistances in histology.

## Conflict of interest

The authors declare that the research was conducted in the absence of any commercial or financial relationships that could be construed as a potential conflict of interest.

## Publisher’s note

All claims expressed in this article are solely those of the authors and do not necessarily represent those of their affiliated organizations, or those of the publisher, the editors and the reviewers. Any product that may be evaluated in this article, or claim that may be made by its manufacturer, is not guaranteed or endorsed by the publisher.

## Abbreviations

α-MEM, Alpha-Minimum essential medium; AKAP-3, A-kinase anchoring protein-3; ALP, Alkaline phosphatase; BCA, Bicinchoninic acid assay; BSA, Bovine serum albumin; ASARM, Acidic serine and aspartate-rich MEPE-associated motif; CPC, Cetylpyridinium chloride; CTX-1, C-terminal telopeptide of type I collagen; DMEM, Dulbecco’s Modified Eagle Medium; ELISA, Enzyme-linked immunosorbent assay; FBS, Fetal bovine serum; FGF-23, Fibroblast growth factor-23; LRP-6, Low-density lipoprotein receptor-related protein 6; MEPE, Matrix extracellular phospho glycoprotein; MCO, Mouse calvarial osteoblast; PBS, Phosphate buffer saline; PHEX, Phosphate-regulating gene with homologies to endopeptidases on the X chromosome; P1NP, Propeptide of type 1 procollagen; PNPP, Para-Nitrophenylphosphate; RANK, Receptor activator of nuclear factor κ B; RUNX-2, Runt-related transcription factor-2; TRAP-5b, Tartrate-resistant acid phosphatase-5b; XLH, X-linked hypophosphatemia; μCT, Micro computed tomography.
