# Supplementary material for: MiR-539-3p impairs osteogenesis by suppressing Wnt interaction with LRP-6 co-receptor and subsequent inhibition of Akap-3 signaling pathway
Source: Front Endocrinol (Lausanne). 2022 Sep 29;13:977347. doi: 10.3389/fendo.2022.977347 (PMC9577939; doi:10.3389/fendo.2022.977347)
Supplement: Supplementary file 1 [file DataSheet_1.pdf]

MiR-539-3p impairs osteogenesis by suppressing Wnt interaction with LRP-6 co-receptor and subsequent inhibition of Akap-3 signaling pathway

Alok Tripathi[1,3], Aijaz A. John, Deepak Kumar[2,3], Saurabh Kumar Kaushal[1,3], Devendra Pratap Singh[1,3], Nazim Husain[1], Jayanta Sarkar[2,3], Divya Singh*[,1,3]

[1]Division of Endocrinology, CSIR-Central Drug Research Institute, Lucknow 226031, India

[2]Division of Cancer Biology, CSIR-Central Drug Research Institute, Lucknow 226031, India

[3]Academy of Scientific and Innovative Research (AcSIR) Ghaziabad, Uttar Pradesh, 201002, India

* **Corresponding author:**

Divya Singh, Division of Endocrinology, CSIR Central Drug Research Institute, Lucknow 226031, India

ORCID ID: 000-0002-2487-4082

Telephone Number: +91-522 2772450 Ext: 4395

Email: divya_singh@cdri.res.in

Supplementary Information

Figure legends

Supplementary Figure 1

Osteoblast cells treated with siAkap3 downregulated the expression of osteoblast differentiation markers. (a) Murine osteoblasts transfected with different concentrations of silencer of *phex* and ALP activity measured at 48 h. Data are expressed in mean ± SEM (n=8). ***P < 0.001 compared between 10 nM NTC (scrambled siRNA), $^{\#\#}$P < 0.01 and $^{\#\#\#}$P < 0.001 compared with 30 nM NTC and $^{\wedge\wedge}$P < 0.01 compared with 50 nM NTC. (b) The expression of osteogenic marker genes (RunX-2, T-1-col, OCN and Osterix) was evaluated in NTC and Akap3 siRNA MCO transfected cells using qRT-PCR. GAPDH used as internal control. Data are expressed as mean ± SEM (n=6) *P < 0.05, **P < 0.01 and ***P < 0.001 compared with NTC. (g) The protein expression of osteogenic markers (RunX-2, T-1-col and OCN) was evaluated in control and Akap3 siRNA MCO treated cells using western blots. (h) Densitometry analysis of blots. All values (mean ± SEM; n=3) * P < 0.05, and **P < 0.01 compared with NTC.

Supplementary Figure 2

miR-539-3p treatment to ovx skeletally mature mice decreased bone formation. (a) Effect of miR-539-3p on osteoblastogenesis markers in bone. All values (mean ± SEM; n=3) * P < 0.05, **P < 0.01 and **P < 0.001 compared with miC group.

Supplementary table -1 Sequences of primers

Serial No.	Gene Name	Primer Sequences
**1.**	GAPDH	F- AGCTTGTCATCAACGGGAAG<br>R-TTTGATGTTAGTGGGGTCTCG
**2.**	ALP	F- CGGATCCTGACCAAAAACC<br>R- TCATGATGTCCGTGGTCAAT
**3.**	Type-1- Collagen	F-CATGTTCAGCTTTGTGGACCT<br>R-GCAGCTGACTTCAGGGATGT
**4.**	BMP-2	F-CGGACTGCGGTCTCCTAA<br>R-GGGGAAGCAGCAACACTAGA
**5.**	RUNX-2	F- GATGATGACACTGCCACCTCT<br>R- AGGGCCCAGTTCTGAAGC
**6.**	OCN	F-TGAGGACCATCTTCTGCTCA<br>R-TGGACATGAAGGCTTTGTCA
**7.**	Osterix	F-TCCCATTCTCCCTCCCTCT<br>R-GGACTGGAGCCATAGTGAGC
**8.**	LRP-6	F-CATGGACATCCAAGTGCTGA<br>R-TTGTCCTCCTCGCATGGT
**9.**	Akap-3	F- AACAGAAAATTACTAAGCACCAACG<br>R- TTGGTGTCTTCACTATCCCTAAGTC
**10.**	TRAP	F-GGTCAGCAGCTCCCTAGAAG<br>R-GGAGTGGGAGCCATATGATTT
**11.**	RANK	F-AGAGGCATTATGAGCATCTCG<br>R-GGAGTGCACTTAGAGGACAGGT
**10.**	mmu-miR-539-3p	CAUACAAGGAUAAUUUCUUUUU
**11.**	siPhex	Sense-GCUGGACCAAGCAACACUCTT<br>Anti sense-GAGUGUUGCUUGGUCCAGCTT
**12.**	silencer Akap3	Sense-CCGCCUCACAAACCUAGUGTT<br>Anti sense-CACUAGGUUUGUGAGGCGGTT

Supplementary table-2 Immunological antibodies used in this study

Antibody	Dilution rate for Western Blot	Species	Company	Cat. No.
**LRP-6**	1:1000	Rabbit	CST	C47E12
**Wnt-3a**	**-**	Rabbit	Abcam	#Ab172612
**GSK3-Beta**	1:1000	Rabbit	CST	9315S
**Beta Catenin**	**-**	Rabbit	CST	8480S
**Phospho-Beta-Catenin**	**-**	Rabbit	CST	9561S
**Lef-1**	1.5000	Goat	Santa Cruz	sc-8591
**RunX-2**	1:1000	Rabbit	Affinity Biotech	AF5186
**BMP-2**	**-**	Rabbit	Affinity Biotech	AF5163
**Type-1-col**	1:1000	Rabbit	CST	91144S
**PHEX**	**-**	Rabbit	Abcam	Ab96072
**Akap-3**	1:1000	Rabbit	Affinity Biotech	AF0529
**Beta-Actin**	1:25000	Mouse	Sigma	A3854
**HRP-conjugated secondary Ab**	1:5000	Anti-Rabbit	CST	7074S
**Anti Goat secondary HRP-conjugated**	1.5000	Anti-Goat	Sigma	AP106P

Lentivirus production

Recombinant lentiviruses, encoding control and target shRNA, were produced in HEK293T stably expressing SV40 large T antigen following standard protocol of three-plasmid lentivirus packaging system. Briefly, 5×10^6 HEK293T cells were plated in 150 mm dish. Overnight grown cells were then transiently transfected through calcium phosphate precipitation method with a combination of 15µg of target AKAP-3 shRNA cloned in pLKO.1-TRC lentiviral vector, 7.5µg each of packaging and envelop plasmid (psPAX2, pMD2.G), 2.5M CaCl2 (100µl) and 2X HBS (1ml). The cell density was kept 40-50% at the time of transfection in order to obtain about 95-100% confluency 72h post-transfection. Two ml of above mixture (AKAP-3 gene shRNA containing pLKO.1-TRC vector, CaCl2, MilliQ and 2X HBS) was mixed properly, incubated at RT for 25 min, then added drop-wise to the plate with media and evenly distributed by swirling the dish. After 6-8 h incubation at 37°C post transfection, media was replaced and the plates were further kept in a CO_2 incubator (at 37°C and 5% CO_2) for additional 48 h. Cell supernatant, containing viral particles, was collected at 48h post transfection and stored at -80°C. Cells were then replenished with fresh growth media and the supernatant was collected again at 72h post transfection. The plates were then bleached and discarded according to approved biosafety procedures. Collected lentiviral supernatant of 48h and 72h was filtered through a 0.45µm low protein binding syringe filter to remove cell debris. Further, PEG8000 (5%) and NaCl (0.15M) were added to the filtered media and the mixture was rotated on a tube-rotor for 16-18h at 4°C to concentrate recombinant lentiviral particles by centrifugation at 12000 rpm for 30 minutes at 4°C.

Transient transduction of cells with lentiviral particles

3T3 cells were plated in a 6 well plate at a seeding density of 0.2×10^6 and grown overnight in a CO_2 incubator. Cells were then transduced with concentrated lentiviral particles, diluted in fresh DMEM-HG media, using cationic polymer polybrene at a final concentration of 10µg/ml. Following day (approximately 22-24 h after transduction) cells were replenished with fresh media. At 72h post-transduction, cells were harvested and processed for immunoblotting to check the knockdown efficacy of the target gene.
