# Supplementary figures and images for: MiR-539-3p impairs osteogenesis by suppressing Wnt interaction with LRP-6 co-receptor and subsequent inhibition of Akap-3 signaling pathway
Source: Front Endocrinol (Lausanne). 2022 Sep 29;13:977347. doi: 10.3389/fendo.2022.977347 (PMC9577939; doi:10.3389/fendo.2022.977347)

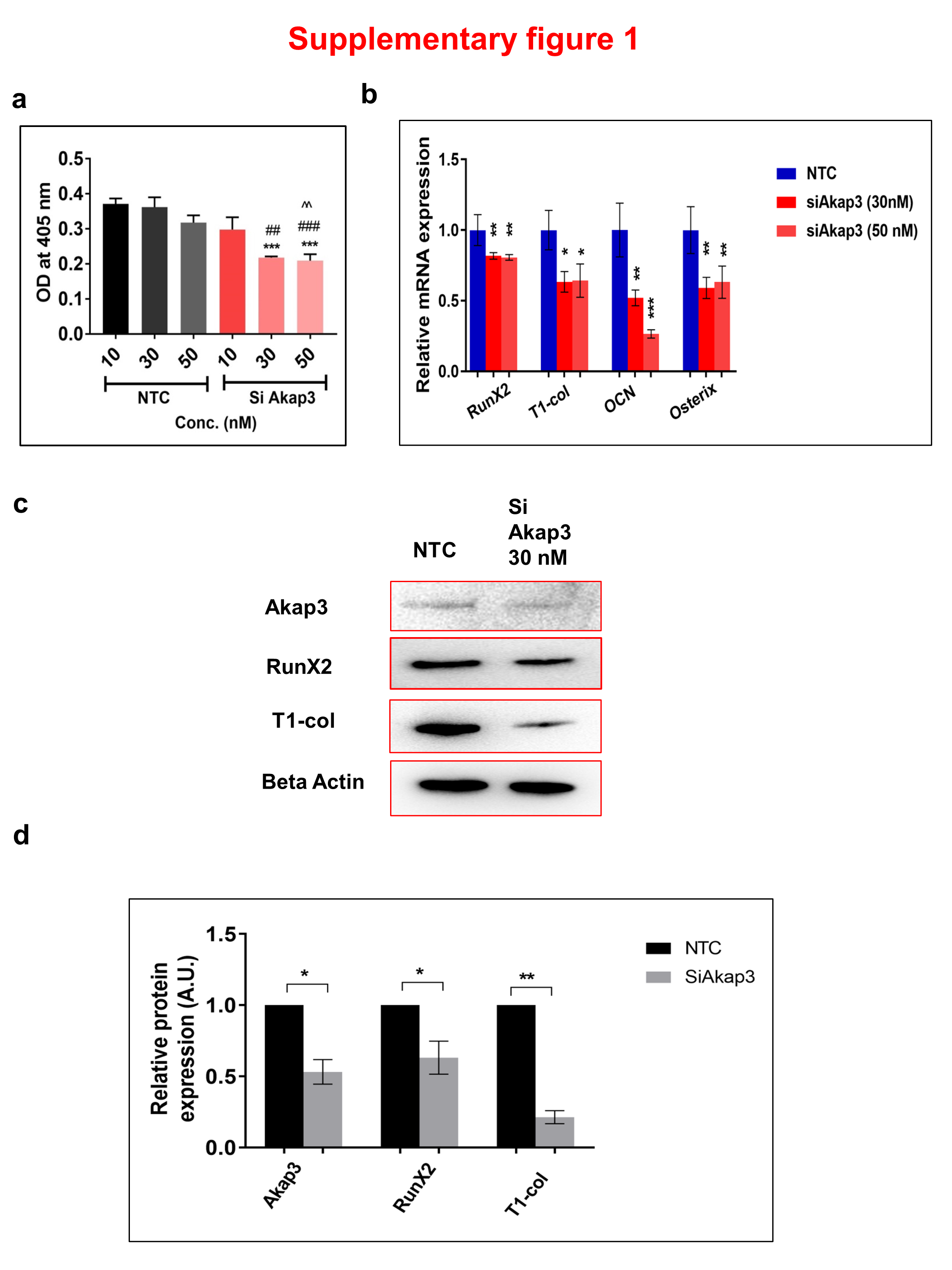

Supplement: Supplementary file 2 [file Image_1.tif]

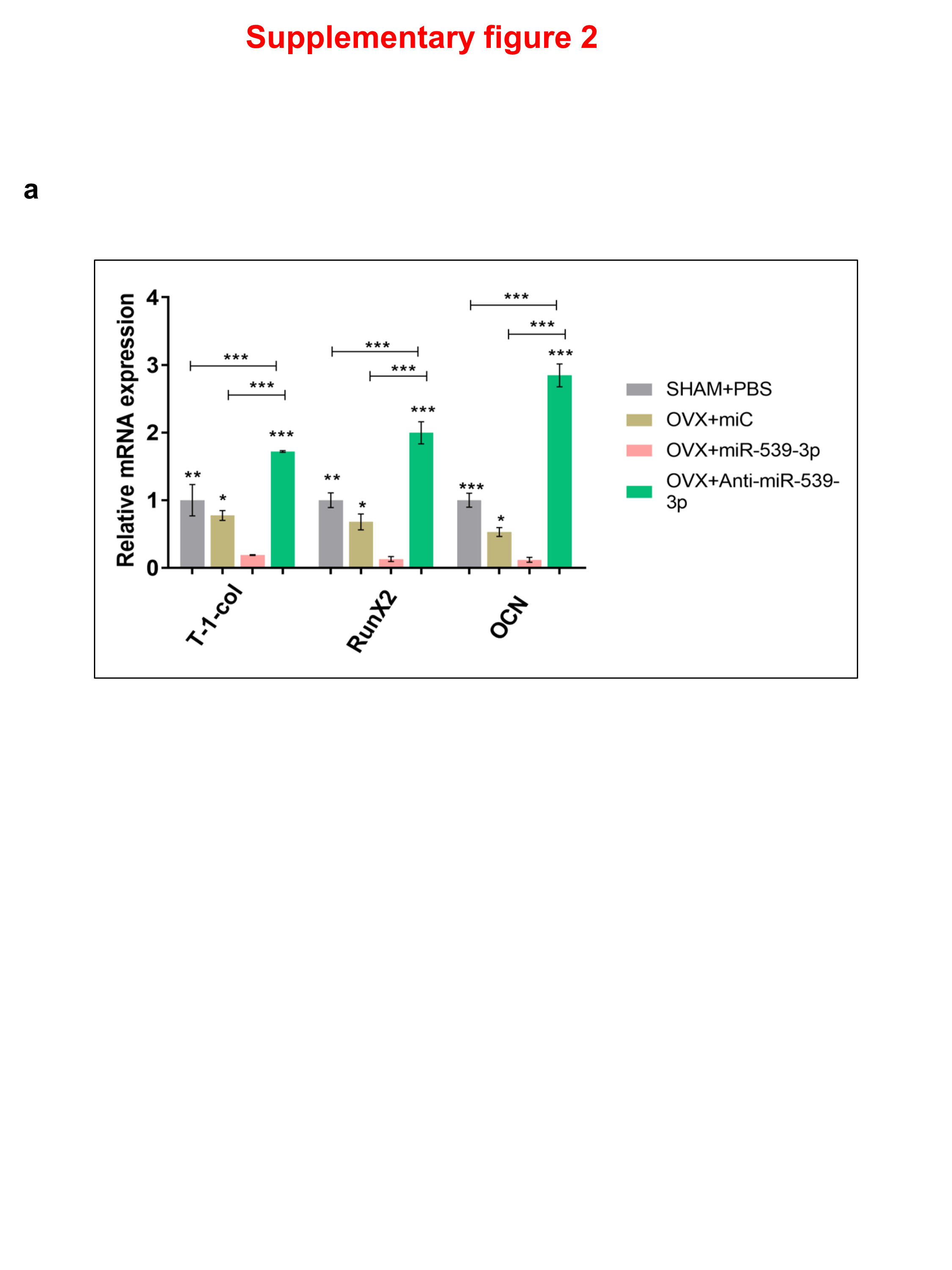

Supplement: Supplementary file 3 [file Image_2.tif]
